# Newborn Infection Control and Care Initiative for health facilities to accelerate reduction of newborn mortality (NICCI): study protocol for a randomized controlled trial

**DOI:** 10.1186/s13063-015-0771-5

**Published:** 2015-06-05

**Authors:** Chivorn Var, Alessandra N Bazzano, Sudesh K. Srivastav, James C Welty, Navapol Iv Ek, Richard A Oberhelman

**Affiliations:** National Institute of Public Health, #2 Kim Y Sung Blvd, Tuol Kork, PO Box 1300, Phnom Pehn, Cambodia; Department of Global Community Health and Behavioral Sciences, Tulane University School of Public Health and Tropical Medicine, 1440 Canal Street, New Orleans, LA 70112 USA; Department of Biostatistics and Bioinformatics, Tulane University School of Public Health and Tropical Medicine, 1440 Canal Street, New Orleans, LA 70112 USA

**Keywords:** Newborn, Infection, Sepsis, Community health, Village health support group

## Abstract

**Background:**

Newborn health is a key issue in addressing the survival of children under five years old, particularly in low and middle income countries, and the evidence base for newborn health interventions continues to evolve. Over the last decade, maternal and under five-year-old mortality and morbidity rates have been successfully reduced in Cambodia, but newborn health has lagged behind. Evidence suggests that an important proportion of newborn mortality both globally and in Cambodia is attributable to infections and sepsis. While initiatives are being implemented to address some causes of newborn illness (related to pre-term birth and asphyxia), a country-level approach to reducing infections has not been formulated. The Newborn Infection Control and Care Initiative (NICCI) is a community and health facility linked intervention to improve health outcomes for newborns.

**Methods/Design:**

The present study applies a cluster randomized trial, using a stepped wedge design, to assess the impact of a package intervention on newborn health. The intervention components include addressing infection control in the perinatal period in health facilities, promoting infection prevention and control practices in health center and home environments, and improving the timeliness of referrals for newborns with suspected infections to appropriate health facilities, by linking families to the medical system through a network of community based volunteers who will make home visits to families in the first week of a newborn’s life.

**Discussion:**

The NICCI trial is designed to complement and enhance the Cambodian Ministry of Health strategies and objectives for maternal and newborn care. Results of the study will help to inform policy and the possible scale-up of newborn health interventions in the country.

**Trial registration:**

This trial was registered with Clinicaltrials.gov (identifier: NCT02271737) on 5 October 2014.

## Background

While global estimates of newborn mortality have been declining, it remains an important public health problem in low income countries, particularly in settings where under five-year-old and maternal mortality rates have decreased more rapidly [1, 2]. It is estimated that up to 40 % of global under five-year-old mortality occurs in the newborn period (during the first 28 days after birth) [3]. In Cambodia, maternal and under five-year-old mortality rates have been significantly reduced over the last 10 years, falling from 472 per 100,000 live births in 2005 to 206 per 100,000 live births in 2010, and 83 per 1,000 live births in 2005 to 54 per 1,000 live births in 2010, respectively. Newborn mortality, however, has been more difficult to address at 28 per 1,000 live births and 27 per 1,000 live births in 2005 and 2010 [4, 5].

In order to address the continued newborn mortality burden, the Cambodian Ministry of Health (MoH), under the guidance of the Prime Minister, resolved to redouble efforts to achieve significant progress in reducing newborn mortality at its Annual Congress in 2011. Attendees at the Annual Health Congress, including the United States Agency for International Development (USAID), the National Reproductive Health Program, and key non-governmental organizations (NGOs) and United Nations partners were called upon and agreed to work with the MoH in reducing high newborn and child mortality rates in Cambodia. As yet, however, there has been no agreed national strategy on how best to achieve the called-for reductions.

The Royal Government of Cambodia prioritized maternal mortality reduction through a fast track initiative, and is committed to improving maternal and child health [6]. While this policy proposes systemic and programmatic improvements directed at the prevention of maternal mortality, it lacks specific strategies for newborn survival in the postnatal period. The persistently high rate of neonatal mortality in Cambodia may be due, at least in part, to the targeting of interventions at the antenatal, delivery, and immediate postpartum periods in the health care facility. Care coordination between the health facility and the community has not been strengthened sufficiently, and infants often receive either inadequate follow-up during the transition from birth facility to community, or no follow-up at all. A total of 63.6 % of mothers reported receiving a postnatal visit within the first four hours after birth, but only 7.4 % were seen by a health care professional four to 23 hours post-delivery, and only 11.3 % were seen in the crucial period of one to two days after the baby was born [5].

A number of trials have been conducted in sub-Saharan Africa and South Asia [7–10] illustrating the utility of home visits by community volunteers to reduce newborn mortality. A recent Cochrane review, on the subject of community based intervention packages for reducing maternal and neonatal morbidity and mortality and improving neonatal outcomes, found encouraging evidence of the value of integrating facility care with newborn care in community settings through a range of interventions delivered via community health workers [11]. While community-level care is important, even greater impact may be achieved by simultaneously improving treatment of ill newborns at the community and health facility level, particularly in cases of infection and sepsis [12]. Improving service delivery for newborns through hygienic facilities and facilitated referral may impact the Cambodian development target for child health.

Currently, clinics follow the Safe Motherhood Clinical Protocol (SMCP) for health centers, which provides standard guidelines for health centers on maternal and newborn care. The SMCP instructs midwives on signs of maternal infection that should be monitored: fever, discharge, and upper or lower urinary tract infections.

The Management of Selected Newborn Problems section of the SMCP provides guidance to the health center staff for providing education to mothers on identifying symptoms and conditions that should prompt the family to bring their newborn to the health facility. However, the protocol is not clear on how to treat some of the noted conditions. For example, parents are advised that convulsions are a danger sign indicating the infant should be referred to the health facility, but there is no guidance for medical providers on how to manage the newborn with convulsions upon arriving at the health facility. The SMCP provides guidance on teaching mothers how to treat some health issues at home, such as treating local infection using gentian violet for skin infection or 1 % tetracycline eye ointment for eye infection, but there is no instruction on what steps to take if there is no improvement [13]. As a result, clinic staff are not properly trained to treat the child, and families may be sent home or resort to the private sector. If home treatment does not work, the newborn is then referred to the provincial or national hospital for a higher level of care.

Formative research conducted in the setting for this study, Takeo province, revealed gaps in essential newborn care practices [14], and barriers to infection prevention and control [15]. The data from that formative research guided the intervention design, and contributed to identifying behavioral and logistical targets for the intervention package components.

### Setting

**Fig. 1:**
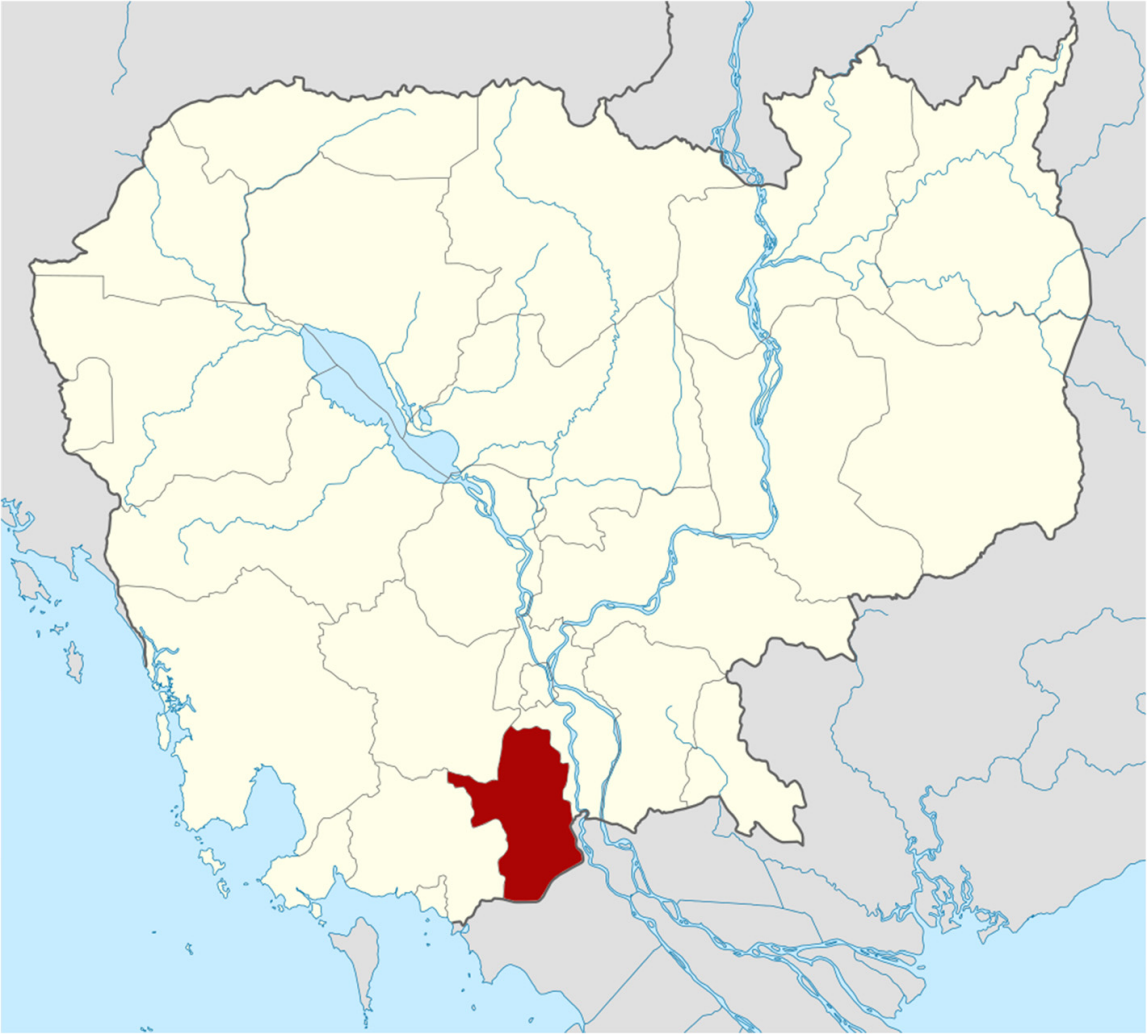
Map showing location of Takeo Province in Cambodia. The study will be conducted in Takeo Province (highlighted in red), which is south of the capital city, Phnom Penh, bordering Vietnam

Takeo is one of 25 provinces in Cambodia, located in southern part of the country, with a population of 843,931 (Fig. 1). The province is subdivided into 10 political administrative districts and 100 communes. As with other provinces, the health system includes a provincial hospital and one-referral hospital for each operational district. In Takeo, there are five operational districts (health administrative districts), 73 health centers, three primary referral hospitals, and one secondary referral hospital [16]. In each village there are at least two Village Health Support Groups (VHSGs), which are made up of community health volunteers.

## Methods/Design

The Newborn Infection Control and Care Initiative (NICCI) is a community and health facility linked intervention to improve health outcomes for newborns. The trial aims to develop and implement a package intervention at the health center, community, and household levels, designed to improve care practices and care-seeking for newborns. The intervention will be evaluated through a cluster randomized trial. The findings of the study will be used to inform policy and strategies affecting newborns in Cambodia.

### Specific objectives

The specific objectives of this trial are as follows:

1. To improve infection control behavior of staff in selected health centers, and train staff on infection control messages to be delivered to pregnant and newly delivered women.
2. To improve referral of sick newborns by VHSG volunteers and health center staff through: telephone-based care coordination between health center staff and VHSG volunteers when newborns are sent home with mothers; increased recognition of danger signs; and shorter time between recognition of danger signs and care-seeking at an appropriate health facility.
3. To improve knowledge of newborn danger signs and appropriate referral for sick newborns by mothers and families of newborn infants.
4. To diagnose the causes of sepsis among infants with possible sepsis who are hospitalized at Takeo Provincial Hospital, through blood cultures and appropriate antibiotic therapy.

### Hypotheses

Our first hypothesis is that by delivering a coordinated intervention that combines improved education for health center midwives, village health care workers, and mothers of newborns, along with improved care coordination via an increased number of interactions (points of contact) between mothers and health care personnel, we will see improved knowledge of both infection prevention methods and newborn danger signs among mothers and health care workers, more rapid case detection of significant newborn illnesses, and more rapid and appropriate referral of ill newborns in the intervention areas, as compared with those not yet receiving the intervention. We also hypothesize that the common causes of newborn sepsis in Cambodia are different from those reported in other regional settings. We will evaluate the causes of newborn sepsis in infants referred to Takeo Provincial Hospital.

### Outcomes

#### Primary outcomes

The primary outcomes for the study are as follows:

1. Percentage of mothers who know at least three danger signs;
2. Percentage of VHSG volunteers who know six danger signs;
3. Percentage of families who seek care from an appropriate facility;
4. Decreased time between onset of suspected danger signs and referral to appropriate facility;
5. Improved infection prevention behaviors by family, as measured by the proportion of hand washing with soap at key events, calculated as a composite score from the percentage of mothers or caretakers reporting hand washing at the following key events: after going to toilet, before touching newborn, before eating, and after cleaning baby’s bottom;
6. Improved infection prevention and control behavior among health center staff, as measured by the proportion of appropriate hand washing with soap or disinfectant at key points in provision of care to mothers and newborns, calculated as a composite score from: percentage of health center staff reporting hand washing: before patient contact (when they examine mother and newborn), before and after aseptic procedure, after exposure to blood or body fluids, after patient contact (after removing gloves), and after touching patient surroundings.

#### Secondary outcomes (intermediate outcomes)

The secondary outcomes for the study are as follows:

1. Percentage of newborns visited at least once by VHSG volunteers on or before day seven of life,
2. Percentage of newborns visited at least twice by VHSG volunteers or before day seven of life,
3. Percentage of VHSG volunteers who can deliver hygiene messages,
4. Percentage of mothers who received messages on hygiene from health center staff,
5. Percentage of mothers who received messages on hygiene from VHSG volunteers,
6. Percentage of mothers who received messages on care-seeking from VHSG volunteers,
7. Percentage of health center staff who know six danger signs, and
8. Percentage of health center staff who recall hygiene messages.

#### Exploratory outcomes

The exploratory outcomes for the study are as follows: all-cause newborn mortality, and cause- and age-specific newborn mortality. While our study may not be sufficiently powered to show reductions in mortality, we anticipate that if the hypothesis above is sustained with larger populations, the intervention will be associated with a reduction in all-cause mortality, and more specifically, in infection-associated mortality.

### Process evaluation

The intervention is likely to reduce the risk of infection, and improve the timeliness of care-seeking if infection does occur. By doing so, neonatal morbidity and mortality would likely be reduced. As this is a package intervention, the indicators chosen will evaluate different components to facilitate understanding of how components related to any change in intervention versus comparison groups. Indicators have been selected which best represent the pathways to determining the effect of the intervention, and additional process indicators will further contribute to understanding the intervention effects and why the intervention was or was not effective. The indicators chosen have also been used in several other comparable studies of package interventions aimed at improving newborn survival at a community level. Reach, coverage and/or delivery exposure, and fidelity will be evaluated during the process evaluation.

#### Primary outcomes

The primary outcomes of the process evaluation are as follows:

1. Percentage of newborns visited by a VHSG volunteer within 24 hours of arriving home,
2. Percentage of newborns visited by a VHSG volunteer on day three of life,
3. Percentage of newborns visited by a VHSG volunteer on day seven of life,
4. Number of VHSG volunteers trained,
5. Number of health center staff trained,
6. Presence of training materials in VHSG equipment, and
7. Presence of training materials in health centers.

### Trial design

The health center functions as the unit of allocation, with health centers randomized to an intervention start date using a stepped wedge design. The design is principally a matched one, with before and after comparisons for each unit of randomization. On the hypothesis that 16 health centers are recruited, one health center will be assigned to start each month, and randomization will be stratified in order to ensure balance across time points. In this design, all clusters (health centers) receive the intervention in due course (unidirectional crossover) and, in particular, the intervention is never removed once it has been implemented, which allows convenience and alleviates ethical concerns. All cases will be used in the primary analysis, constituting an intent-to-treat approach. Full study enrollment will be completed at 16 months and duration of participation in the intervention will vary based on randomization assignment, with all clusters enrolled with a unidirectional approach and participating for the last six months of the trial.

All health centers in the trial will be included in the intervention, but will be randomized to one of 16 start dates. The center-level intervention will be implemented one stage at a time, progressively over the study period, until all centers have received the intervention. In addition to center level, patient-level data will also be collected. Thus the analysis is divided into center level and patient level, with the patient-level analysis as the primary outcome. All live births occurring in the study area will be eligible, and pregnant women in the last trimester will be recruited from health center catchment areas using the stepped wedge design, where each area will serve initially as control and later as intervention. The study will enroll a target sample size of 1,957 recently delivered women.

### Data collection

Informed consent will be sought from all participants prior to enrollment. The health-center-level baseline data will be collected from all 16 clusters, by health center coordinators using an environmental assessment checklist, prior to the start of the intervention. The same tool will be used throughout intervention implementation and at the conclusion of activities. World Health Organization guidelines indicate that a simple checklist to assess infection prevention control practice, using key indicators, can provide important information for process monitoring and evaluation [17]. Individual-level data related to the outcomes noted above, including primary, secondary, and exploratory outcomes, will be collected through interview by VHSG project coordinators during enrollment in the third trimester of participant’s pregnancy, and on the 14th and 29th day of newborn life, at the household level.

Midwives will provide mothers with a referral form at the time of discharge of the newborn from the health center (the day after birth), in case the infant is subsequently brought to the hospital for any reason. The form will alert hospital staff to collect a blood culture on arrival for infants presenting with possible infectious diseases prior to administering antibiotics. Infants under one month of age presenting to facility with signs of sepsis, based on the definitions given by Zaidi *et al*. [12], will have blood samples taken for culture and assayed at Takeo hospital laboratory.

### Sample size and power calculation(s)

Using all-cause newborn mortality as the exploratory outcome and a stepped wedge design for cluster randomized trials, we would require a total sample size of 1,957, based on 80 % power, a 5 % significance level, and a 10 % rate of loss-to-follow-up, to detect a 20 % reduction in neonatal mortality with a baseline of up to 40 deaths per 1,000 live births at the provincial level. For this study, health centers with an expected minimum of 20 births per month will be included, resulting in a study population of at least 320 per month, or 5,540 participants over one year and five months. This will allow us to meet more than 80 % power of the study primary objectives. Secondary analyses will examine the impact through process indicators based on a smaller sample. Other intervention phase outcomes with higher baseline rates than all-cause mortality (incidence of newborns with specific danger signs and proportion of newborns with danger signs referred to an appropriate facility) are hypothesized to demonstrate 20 % or greater improved outcome in the intervention group, but the lack of reliable baseline data on these does not allow us to use them for power calculations.

### Analysis plan

The analyses of study data will be performed using the principle of intention-to-treat for the analysis of randomized controlled trials. The exploratory data analysis will be performed for all descriptive statistics, such as means, proportions, variances, and correlation by time and group. Two group (control and intervention) comparison analysis at each time point, across all time points and within each cluster will be performed. One-way crossover design analysis methods will be used for stepped wedge design data, that is, analyses will be performed accounting for potential within-cluster correlation using a random effect model, multilevel model, or generalized estimating equations. Based on each month of the study, we will compare the neonatal mortality rate in those centers that have not yet received the intervention with those in the centers that have. The study hypothesis will be tested on the significance level of alpha = 5 % throughout the analysis. All analyses, summaries, listing, and graphing will be performed on the data, using SAS software (version 9.1 or higher in a Windows environment SAS Institute Inc. Cary, North Carolina, USA). In order to avoid the potential problem of multiple comparisons related to the primary outcomes we plan to apply a Bonferroni correction.

### Capacity development

From the implementation study, we envisage the following capacity development at the provincial level:

1. Through training and supervision, the intervention health centers will learn a simple approach in improving newborn care based on their existing resources, such as infection prevention control practices, and coordination and follow-up of newborns with VHSGs.
2. Through training and supervision, VHSGs in the intervention clusters will be equipped with better knowledge and effective educational materials for providing health education on newborn health, and will understand and be able to help mothers and families to act faster on newborn referral.
3. Through meetings and updates on the progress of the study, the management of the Takeo Provincial Health Department will be informed and understand the implementation modality of the study.

We envisage the following capacity development at the national level:

1. The National Maternal and Child Health Center (NMCHC), whose director is a member of the Study Advisory Committee, will contribute to the operational modality of the study through sharing the study’s annual progress report, informal interaction with the project staff, and periodic update during NMCHC sub-technical working group meetings.
2. Stakeholders in-country, such as USAID/Cambodia, other United Nations agencies, and NGOs such as Reproductive Health Association of Cambodia (RHAC), Reproductive and Child Health Alliance (RACHA), University Research Corporation (URC), Cooperative for Assistance and Relief Everywhere (CARE) International, and so forth will be apprised of intervention progress through participation in the sub-technical working groups and access to the study publications.
3. The National Institute of Public Health, through the management of the study, will be able to further improve the capacity of the faculty and administrative staff, for example in research, grant management and compliance with trial requirements.

### Data dissemination plan and knowledge transfer

Research results will be shared with all study stakeholders, and promptly discussed with the local health center and village-level participants. Local dissemination meetings with the study populations will be held, including through presentation of the study during Operational Health District monthly meeting and during Provincial Technical Working Group meetings. After the intervention has concluded, a detailed evaluation report of lessons learned will be distributed to relevant partners, along with any intervention materials of interest. Policy briefs will be circulated nationally and internationally to relevant organizations.

Dissemination of study results will also occur at scientific meetings and through publications on the impact of the intervention on neonatal morbidity and mortality rates, behavior change, impact on neonatal care in facilities, referral process and follow-up of neonates, impact on infection control, types of pathogens isolated from neonates clinically diagnosed with sepsis, process outcomes, and lessons learned concerning working with health center providers and VHSG volunteers, supervision, monitoring, and performance; training on danger signs; and social or cultural factors effecting response to specific care recommendations including special care for low birth weight babies and referrals.

It is anticipated that dissemination will result in improved policies and strategies to reduce newborn mortality in Cambodia, especially given the central role of the NMCHC (who determine MNCH national policy) and NIPH in the conduct of the study. The Study Advisory Committee will also facilitate dissemination and integration of translational findings. Study partners at the national level are committed to translating research findings into policy and documenting promising intervention practices for further scale-up. NGO partners supporting other health centers will also be invited to share the results and benefit from technical support from the investigators.

### Ethical approval

The study has been approved by Federalwide Assurance (FWA)-certified institutional review boards (IRBs) in Cambodia and the United States. In Cambodia, the responsible IRB is IRB00003143 - National Ethics Committee Health Research IRB #1 under FWA 00017450 National Institute of Public Health. In the United States, the responsible IRB is IORG0000197 - Tulane University Medical Center under FWA 00002055 Tulane University, with approval through an Institutional Review Board Authorization Agreement.

## Discussion

This study addresses the need to evaluate newborn health approaches linking behavior change in the health center and home environment with improved care-seeking at the community level. The intervention will provide data on how infection prevention control, improved linkages between health centers and community volunteers, and prompt care-seeking will impact newborn health. The results of this study will provide data for policy-level actions on newborn survival in Cambodia, as well as in other settings with similar health outcomes. It will also contribute to the potential scale-up of a linked model of community-facility care for newborns.

### Trial status

This study is currently in the early stages of implementation. Recruitment began 19 February 2015.

## Abbreviations

NICCI: Newborn infection control and care initiative for health facilities to accelerate reduction of newborn mortality
NMCHC: National Maternal and Child Health Center
SMCP: Safe motherhood clinical protocol
VHSG: Village health support group
